# Supplementary material for: The Prognosis of Baseline Mitral Regurgitation in Patients with Transcatheter Aortic Valve Implantation
Source: J Clin Med. 2021 Sep 2;10(17):3974. doi: 10.3390/jcm10173974 (PMC8432060; doi:10.3390/jcm10173974)
Supplement: Supplementary file 1 [file jcm-10-03974-s001.zip › jcm-1351759-supplementary.pdf]

Supplementary Materials

Table S1. Baseline characteristics of patients treated with TAVI according to baseline tricuspid regurgitation.

	No Tricuspid Regurgitation	Tricuspid Regurgitation	*p*	Total
	**(*n* = 41,045)**	**(*n* = 1821)**		**(*n* = 42,866)**
Age, years	82.8 ± 6.7	82.1 ± 7.6	0.0001	82.7 ± 6.8
Sex (female)	21033 (51.2)	936 (51.4)	0.9	21969 (51.3)
Charlson comorbidity index	4.0 ± 2.8	4.6 ± 2.8	<0.0001	4.0 ± 2.8
Frailty index	5.4 ± 5.7	6.5 ± 6.1	<0.0001	5.4 ± 5.8
EuroSCORE II	3.7 ± 1.0	4.0 ± 1.1	<0.0001	3.7 ± 1.0
Hypertension	33008 (80.4)	1528 (83.9)	0.0002	34536 (80.6)
Diabetes mellitus	12081 (29.4)	515 (28.3)	0.29	12596 (29.4)
Heart failure with congestion	22845 (55.7)	1338 (73.5)	<0.0001	24183 (56.4)
History of pulmonary edema	2085 (5.1)	164 (9.0)	<0.0001	2249 (5.2)
Aortic regurgitation	4644 (11.3)	493 (27.1)	<0.0001	5137 (12.0)
Mitral regurgitation	6978 (17.0)	1262 (69.3)	<0.0001	8240 (19.2)
Previous endocarditis	270 (0.7)	27 (1.5)	<0.0001	297 (0.7)
Dilated cardiomyopathy	6402 (15.6)	501 (27.5)	<0.0001	6903 (16.1)
Coronary artery disease	25112 (61.2)	1241 (68.1)	<0.0001	26353 (61.5)
Previous myocardial infarction	5776 (14.1)	302 (16.6)	0.003	6078 (14.2)
Previous PCI	11895 (29.0)	538 (29.5)	0.6	12433 (29.0)
Previous CABG	3412 (8.3)	215 (11.8)	<0.0001	3627 (8.5)
Vascular disease	14773 (36.0)	881 (48.4)	<0.0001	15654 (36.5)
Atrial fibrillation	18167 (44.3)	1208 (66.3)	<0.0001	19375 (45.2)
Previous pacemaker or ICD	8304 (20.2)	525 (28.8)	<0.0001	8829 (20.6)
Ischemic stroke	2233 (5.4)	96 (5.3)	0.76	2329 (5.4)
Intracranial bleeding	606 (1.5)	30 (1.6)	0.55	636 (1.5)
Smoker	3121 (7.6)	227 (12.5)	<0.0001	3348 (7.8)
Dyslipidemia	19072 (46.5)	958 (52.6)	<0.0001	20030 (46.7)
Obesity	10324 (25.2)	571 (31.4)	<0.0001	10895 (25.4)
Alcohol related diagnoses	1712 (4.2)	107 (5.9)	0.0004	1819 (4.2)
Abnormal renal function	7035 (17.1)	451 (24.8)	<0.0001	7486 (17.5)
Lung disease	9631 (23.5)	531 (29.2)	<0.0001	10162 (23.7)
Sleep apnea syndrome	3605 (8.8)	203 (11.1)	0.001	3808 (8.9)
COPD	6080 (14.8)	345 (18.9)	<0.0001	6425 (15.0)
Liver disease	1956 (4.8)	152 (8.3)	<0.0001	2108 (4.9)
Gastroesophageal reflux	1383 (3.4)	76 (4.2)	0.06	1459 (3.4)
Thyroid diseases	5598 (13.6)	358 (19.7)	<0.0001	5956 (13.9)
Inflammatory disease	4112 (10.0)	247 (13.6)	<0.0001	4359 (10.2)
Anemia	11163 (27.2)	665 (36.5)	<0.0001	11828 (27.6)
Previous cancer	7646 (18.6)	366 (20.1)	0.12	8012 (18.7)
Edwards Sapien XT	4096 (10.0)	136 (7.5)	0.0004	4232 (9.9)
Edwards Sapien 3	20725 (50.5)	917 (50.4)	0.91	21642 (50.5)
Medtronic Corevalve	5090 (12.4)	185 (10.2)	0.004	5275 (12.3)
Medtronic Evolut	11134 (27.1)	583 (32.0)	<0.0001	11717 (27.3)
Self-expandable TAVI	16224 (39.5)	768 (42.2)	0.02	16992 (39.6)
Balloon-expandable TAVI	24821 (60.5)	1053 (57.8)	0.02	25874 (60.4)

Values are *n* (%) or mean ±SD. CABG: coronary artery bypass graft; COPD: chronic obstructive pulmonary disease; ICD: Implantable Cardioverter Defibrillator; PCI: percutaneous coronary intervention; TAVR: transcatheter aortic valve implantation.

Table S2. Clinical outcomes in patients treated with TAVI according to baseline mitral regurgitation**.**

	**No MR (*n* = 34,626)**	**MR (*n* = 8240)**		
	Incidence, %/Year (95%CI)	Incidence, %/Year (95%CI)	Hazard Ratio (95%CI)	*p*
All-cause death	14.39 (14.02–14.78)	16.89 (16.07–17.75)	1.192 (1.125–1.263)	<0.0001
Cardiovascular death	6.28 (6.03–6.53)	8.23 (7.66–8.83)	1.313 (1.210–1.425)	<0.0001
Stroke	3.20 (3.02–3.39)	3.16 (2.81–3.55)	0.988 (0.868–1.124)	0.85
Rehospitalization for heart failure	17.73 (17.29–18.20)	25.29 (24.18–26.45)	1.411 (1.340–1.486)	<0.0001
	**No TR (*n* = 41045)**	**TR (*n* = 1821)**		
	Incidence, %/year (95%CI)	Incidence, %/year (95%CI)	Hazard Ratio (95%CI)	*p*
All-cause death	14.73 (14.38–15.08)	18.75 (16.85–20.87)	1.272 (1.140–1.419)	<0.0001
Cardiovascular death	6.54 (6.31–6.78)	9.52 (8.19–11.06)	1.396 (1.196–1.630)	<0.0001
Stroke	3.19 (3.03–3.36)	3.32 (2.56–4.29)	1.029 (0.792–1.338)	0.83
Rehospitalization for heart failure	18.77 (18.34–19.20)	29.28 (26.64–32.18)	1.509 (1.369–1.663)	<0.0001

Values are *n* (yearly incidence rate, %). HR: hazard ratio.

A

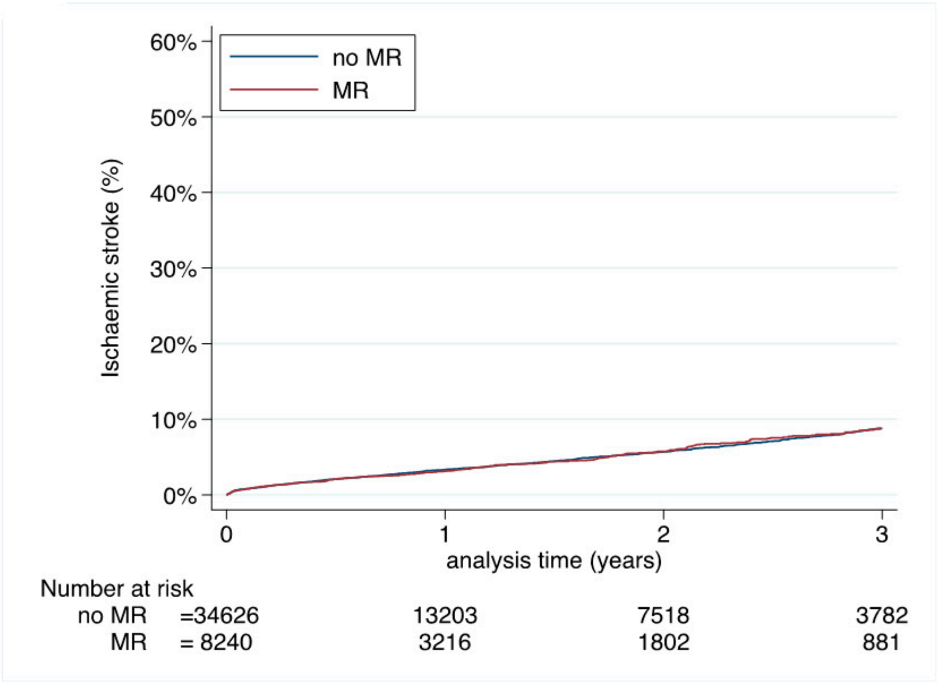

B

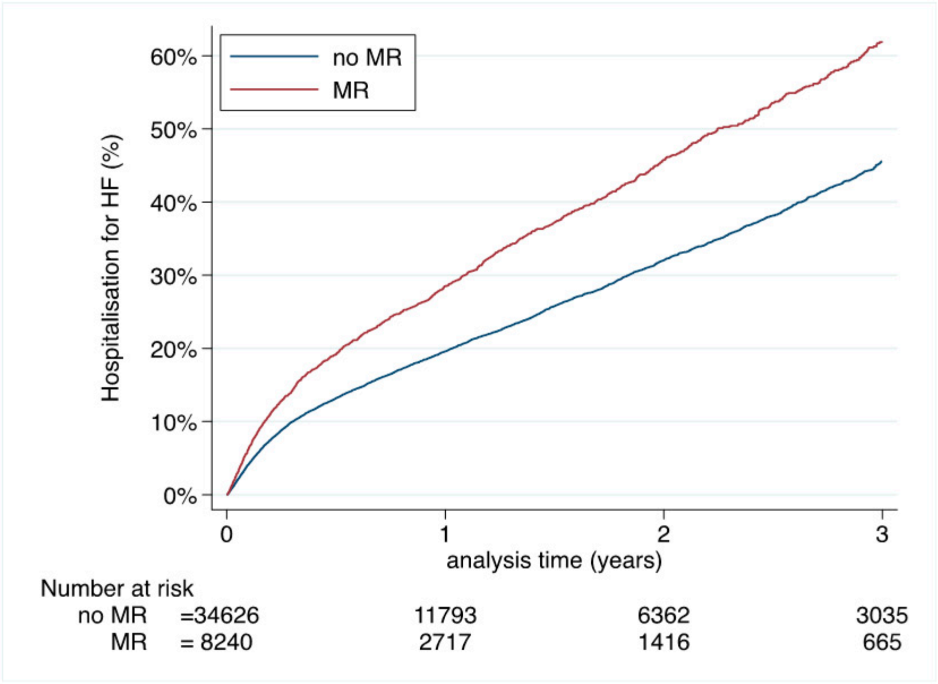

Figure S1. Cumulative incidence for ischemic stroke (top panel, **A**) and hospitalization with heart failure as first diagnosis (lower panel, **B**) in patients treated with TAVI with MR or no MR at baseline.

A

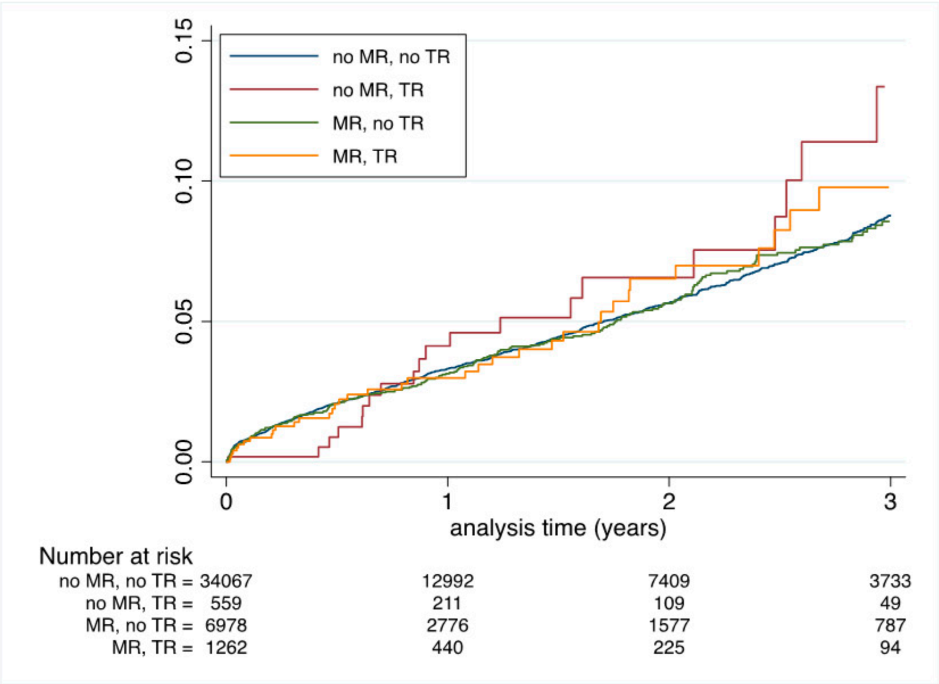

B

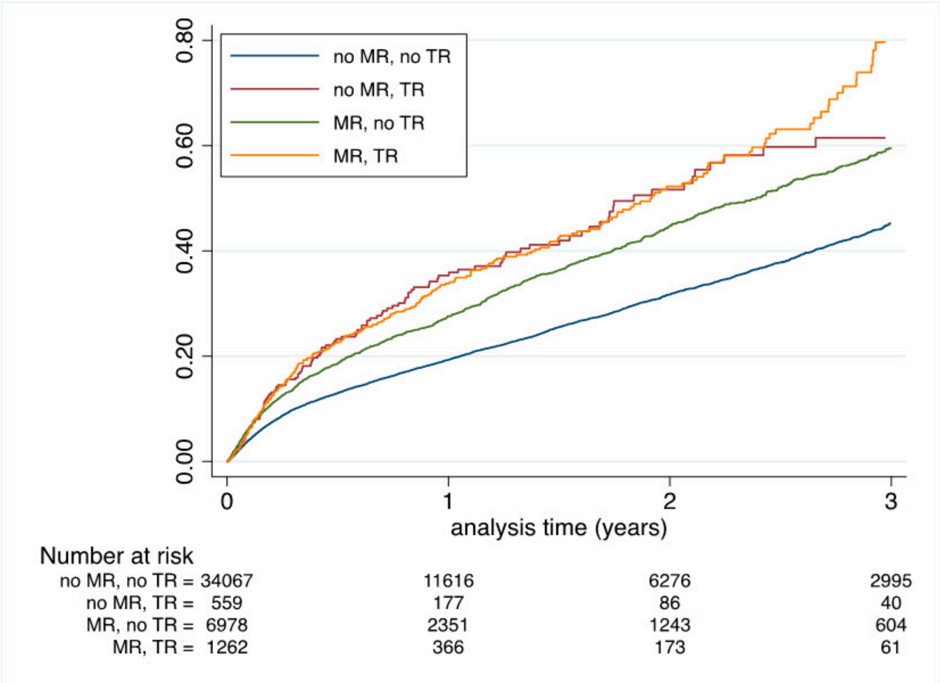

Figure S2. Cumulative incidences for ischemic stroke (top panel, **A**) and re-hospitalization for heart failure (lower panel) of patients treated with TAVI according to MR (or no MR) and TR (or no TR, **B**) at baseline.
